# Effect of hypoxic conditioning on functional fitness, balance and fear of falling in healthy older adults: a randomized controlled trial

**DOI:** 10.1186/s11556-021-00279-5

**Published:** 2021-12-01

**Authors:** Rafael Timon, Marta Camacho-Cardeñosa, Adrián González-Custodio, Guillermo Olcina, Narcis Gusi, Alba Camacho-Cardeñosa

**Affiliations:** 1 Faculty of Sport Sciences, University of Extremadura, Cáceres, Spain; 2 Faculty of Education, University of Nebrija, Madrid, Spain

**Keywords:** Older adults, Hypoxia, Strength training, Fitness, Falling

## Abstract

**Background:**

Hypoxic conditioning has been proposed as a new tool to mitigate the sarcopenia and enhance health-related function, but decrements in standing balance have been observed during hypoxia exposure. The aim of the study was to evaluate the effect of a hypoxic conditioning training on functional fitness, balance and fear of falling in healthy older adults.

**Methods:**

A total of 54 healthy older adults (aged 65–75 years), who voluntarily participated in the study, were randomly divided into three groups: the control group (CON), the normoxia training group (NT) that performed strength training in normoxia, and the hypoxia training group (HT) that trained under moderate hypoxic conditions at a simulated altitude of 2500 m asl. The training programme that was performed during 24 weeks was similar in both experimental groups and consisted of a full-body workout with elastic bands and kettlebells (three sets × 12–15 reps). The Senior Fitness Test (SFT), the Single Leg Stance test (SLS) and the Short Falls Efficacy Scale-International (FES-I) were assessed before and after the intervention.

**Results:**

Results showed that after training, either in normoxia or in hypoxia, the participants increased upper and lower body strength, and the aerobic endurance, and decreased the fear of falling.

**Conclusions:**

The moderate hypoxic conditioning seems to be a useful tool to increase the functional capacity in healthy older adults without observing a decline in balance.

**Trial registration:**

ClinicalTrials.gov NCT04281264. Registered February 9, 2019-Retrospectively registered.

## Background

Aging, even in the absence of chronic disease, is associated with a variety of biological changes that can contribute to decreased skeletal muscle mass and strength [1, 2], as well as a loss of physical and cognitive functionality [3, 4]. Additionally, the loss of functional capacity and muscular strength in the lower body are considered responsible for a greater risk of falls [5]. All these unhealthy changes could be attributed to a variety of causes, such as disuse, alteration of protein synthesis, chronic inflammation or increase in inter and intramuscular adipocyte content [6, 7].

In general, physical exercise has been recognized as a fundamental tool in the treatment and prevention of chronic diseases associated with aging [8]. More specifically, resistance training and multifactorial physical training are effective at improving the functional capability and quality of life of the older people, as well as attenuating the loss of muscle mass and reducing the risk of falls associated with aging [9–11]. However, not all strength training methods are a viable option for older people, being necessary to adjust factors such as intensity, volume, frequency and type of exercises [7]. In this sense, strength training with elastic bands has been proposed as a safe, low-cost and effective method to promote significant beneficial effects in older people [12, 13].

However, new methods and protocols are being studied to optimize the benefits and physiological adaptations caused by training. Hypoxia conditioning has been proposed as a new therapeutic modality to mitigate the sarcopenia and loss of strength during ageing [14, 15]. Exercise training in hypoxia enhanced health-related function in obese men [16], and an intermittent hypoxia training had positive effects on hemodynamics, microvascular endothelial function, and work capacity of untrained senior men [17]. Even, Schega et al. [18] claimed that hypoxic training is more effective than solely strength-endurance training on cognitive performance and quality of life of older people.

Previous studies have shown that physical conditioning in normoxia, compared to that performed in hypoxia, does not lead to the same acute and/or chronic responses at the cardiovascular and muscular level [19]. In situations of low oxygen availability, the hypoxia-inducible factor 1α (HIF-1α) stabilizes and activates the transcription of genes, some of them involved in processes such as cell survival, angiogenesis or the regulation of the inflammatory response [20]. In addition to this, the resistance training under moderate hypoxia promotes the skeletal muscle cell growth [21] and the motor unit recruitment [22]. However, decrements in postural control and standing balance during hypoxia exposure due to alterations in processing of sensorimotor signals (visual, vestibular, and proprioceptive) within subcortical or cortical structures have also been observed [23, 24]. This fact could have a negative effect on the functional capability of the older adults, although balance was only disrupted during normobaric hypoxia at fraction of inspired oxygen (FIO2) less than 15% [24]. Moreover, while the isolated negative effects of hypoxia on postural balance have been analyzed before, no study has examined the combined effects of normobaric hypoxia and strength training on this parameter in older adults.

Therefore, the aim of this study was to analyze whether a strength training under moderate normobaric hypoxia has beneficial effects on the functional fitness of healthy older adults and the fear of falling, without negatively affecting balance. The starting hypothesis is that the training program will improve the functional capability of the older adults and decrease the fear of falling due to an increase in muscle strength.

## Methods

### Participants

A total of 54 healthy older adults aged 65–75 years participated in the study. The control variables and anthropometric characteristics of the participants are shown in Table 1. Various associations of retired people, nursing homes and senior universities were contacted to inform them about the project and recruit subjects. Participants were selected after a screening visit, in which the following inclusion criteria had to be met: (1) women and men aged 65 years or older, (2) absence of participation in any other type of intervention based on physical exercise in the last 6 months, (3) subjects have not been above 1500 m during the last 3 months, (4) no current medical condition that is not compatible with resistance exercises, and (5) consumption of no more than two alcoholic beverages per day. Group adherence to training was set at 75% attendance. Additionally, they were asked to continue with their usual lifestyle and diet throughout the intervention, and they were allowed to continue using their usual medication. The volunteers, who finally met the inclusion criteria, were divided into three groups in a controlled and randomized design, although balancing the groups by sex: Control group (CON; *n* = 19) that did not perform physical exercise and were instructed to continue with their normal daily activities; Normoxia training group (NT; *n* = 18) that performed a strength training in normoxia; and Hypoxia training group (HT; *n* = 17) that trained at a simulated altitude of 2500 m asl. The research was approved by the Bioethics Committee of the university (Ref: 65/2018) and was carried out respecting the ethical principles established in the Declaration of Helsinki. The participants signed an informed consent and could leave the research at any time.

**Table 1:** Control variables and anthropometric characteristics of the participants before the intervention (mean ± SD)

Groups	Years	Weight (kg)	Height (m)	BMI	Kcal/day	PAR-Q (0–7)
Control (*n* = 19)	70.55 ± 4.0	66.1 ± 10.2	1.56 ± 0.9	26.8 ± 2.6	1941.5 ± 306.1	2.75 ± 0.85
NT (*n* = 18)	70.35 ± 3.3	70.9 ± 11.5	1.61 ± 0.8	27.1 ± 3.9	1847.0 ± 442.6	2.52 ± 1.46
HT (*n* = 17)	68.46 ± 3.8	71.9 ± 14.9	1.64 ± 0.9	26.4 ± 3.3	2004.0 ± 254.55	2.30 ± 0.85
*P*	.508	.430	.601	.892	.697	.589

*NT: Normoxia training group*

*HT: Hypoxia training group*

*BMI: Body mass index*

*PAR-Q: Physical activity rating scale*

### Strength training

The intervention programme was performed by certified trainers. The design of the exercise programme was carried out following the recommendations of the American College of Sport Medicine [25] and lasted 24 weeks, with a training frequency of 3 days per week (Monday, Wednesday and Friday). Before starting the training programme, two familiarization sessions were held with the participants to learn the technique of the exercises and to define the training load. The intensity of the exercises was controlled from the rate of perceived effort (RPE) measured with a visual analogue scale validated for strength exercises [26]. This scale quantifies effort from 0 (extremely easy) to 10 points (extremely hard), and throughout the intervention the subjects had to train with an effort that ranged between 6 and 8.

The duration of the training session was approximately 45 min, which included a 10-min warm-up with body mobilization and dynamic stretching, a 30-min main part and a 5-min cool down with stretching exercises and relaxation. The main part of the session consisted of a circuit in which nine exercises were performed to train several body areas (pectoral, shoulders, back, arms, thighs, legs and abdominals) with a structure of three series of 12–15 repetitions per exercise, with a 1-min rest between sets. Six exercises were performed using elastic bands (chest press, back row, biceps curl, triceps pushdown, standing lateral raise and front shoulder raise), regulating the intensity according to the colour of the band. The tension of the bands varied from low to high depending on the colour of the band (orange/green/blue). Each subject adjusted the resistance to training depending on the grip and the distance reached during the movement. The workload in all the groups was reviewed every 2 weeks throughout the training programme in order to train progressively and to encourage a change in the colour of the band until reaching blue (although always with an effort that ranged between 6 and 8 of RPE). Additionally, two other exercises were developed (squat and lying hip raise) with kettlebells, with loads that evolved from 4 kg to 10 kg. The last exercise was a front plank in isometric conditions for 15 s (and evolving up to 30 s throughout the training), which was repeated three times with a 1-min rest between sets.

Depending on the experimental group, the training was performed in normoxia or in normobaric hypoxia. The NT group trained in a room at 459 m asl. The HT group trained inside a hypoxia chamber (CAT 310, Colorado Altitude Training, USA) located in the training room. The hypoxic environment was produced by a hypoxic generator (CAT 12, Colorado Altitude Training, USA). It was set a FiO2 of 16.1% to simulate an altitude of 2500 m asl. The simulated altitude was calculated according to the chart and guidelines provided by the hypoxic generator manufacturer. FiO2 was controlled regularly with an electronic device (HANDI +, Maxtec, USA). For safety reasons, during the training session in hypoxia, a pulse oximeter was used to ensure that blood oxygen saturation (SpO2%) did not fall below 85%.

### Measurements

On their first visit to the laboratory, subjects answered an adapted version of the Physical Activity Rating Questionnaire (PAR-Q) to evaluate their level of physical activity [27], with scores between 0 (lowest level) and 7 (highest level). Likewise, the caloric intake of the participants was estimated using a 7-day diet inventory, which was analyzed using the diet software Nutriber (Nutriber v1.1.1, Funiber, Spain). Body mass and height was also measured using a portable stadiometer (Seca 213, Germany), and body mass index (BMI) was calculated from the ratio of weight/height^2^ (kg/m^2^).

Body composition variables such as fat mass and lean body mass were calculated before and after the intervention using dual-energy X-ray absorptiometry (DXA, Norland Excell Plus, Norland Inc., USA). The standard CVs was 1.4% for fat mass and 0.9% for lean body mass. The same technician performed all the scans, which were analyzed by a graphical user interface to Windows XP operating system.

Functional fitness was evaluated with the Senior Fitness Test (SFT) [28] by the same technician in the morning (10:00–12:00a.m), before and after 24 weeks of intervention This test battery is considered a reliable instrument to assess functional capability in older adults (≥ 60 years of age) [29]. Participants had to perform six test: chair stand test (to assess lower body strength); arm curl test (to measure upper body strength); 6-min walk (to assess aerobic endurance); chair sit and reach test (to assess lower body flexibility); back scratch test (to assess upper body flexibility); and 8-ft up and go test (to assess agility and dynamic balance).

The single leg stance (SLS) test was used to assess static postural and balance control. SLS test a valuable tool for monitoring postural disturbance and musculoskeletal status as well as for managing fall risk [30]. The test was performed with eyes open and hands on the hips. Participant had to stand unassisted on dominant leg, timed from the time the other foot leaves the ground till when the foot touches the ground again or the arms leave the hips. A maximum time of 45 s was set for the time recording.

An assessment of fear of falling was also made. Participants had to complete the Short Falls Efficacy Scale-International (FES-I) questionnaire [31], before and after the intervention. This questionnaire consists of a scale that ranges from a minimum value of 7 points (without fear of falling) to a maximum of 28 points (severe fear of falling).

Control of effort during training sessions was monitored weekly in all subjects. SpO2%, heart rate (HR) and RPE were measured. Measurements were made 3 min after the last exercise of the session had ended. The mean values obtained from the 24 weeks that the intervention lasted were calculated. SpO2% was measured in duplicate using a pulse oximeter (Wristox 3100; Nonin, USA). HR was monitored using a HR monitor (Polar Z9, Finland) and beats per minute (bpm) were recorded. RPE was obtained by showing a graphical scale to participants with a category-ratio 10 scale, ranging between 1 (extremely easy) and 10 (extremely hard) [26].

### Statistical analyses

Statistical analyses were performed using the Statistical Package for Social Sciences (SPSS, version 23.0, Chicago, IL, USA). The Shapiro-Wilks test was applied in order to verify a normal distribution of data, and Levene’s test was used to assess the homogeneity of variance. A repeated measures ANOVA was performed for each variable to explore within-group and between-group differences over time, using the baseline values as covariates. Post hoc Bonferroni tests were performed when appropriate using the SPSS syntax commands. The confidence interval (CI) for the difference between pre-post has been shown to provide an estimate of the absolute difference in means of variables of interest. The effect size (ES) and the percentage change (%∆) from Pre- to Post- were also calculated when there were significant differences. The magnitude of effect was classified as trivial (0.25), small (between 0.25 and 0.50), moderate (between 0.50 and 1.0) and large (> 1.0). The significance level was set at *p* ≤ 0.05, with a confidence level of 95%.

## Results

A total of 54 healthy older adults completed the intervention, and their results were included in the analysis. There were no research-related adverse effects or injuries. The control variables of the participants are shown in Table 1. Prior to the intervention, no significant differences between groups were observed for any variable.

The SpO2% values shown in Table 2 indicate that the environmental conditions during training sessions were different between NT and HT. SpO2% was significantly lower (*p* = 0.001) during HT than in NT. No differences were observed between groups with regard to HR and RPE.

**Table 2:** Variables measured during resistance training sessions (mean ± SD)

Variables	NT	HT	*p*
SpO2 (%)	96.2 ± 1.8	90.0 ± 1.4	**.001††**
HR (bpm)	87.4 ± 14.7	96.5 ± 17.5	.370
RPE	5.8 ± 0.7	5.9 ± 0.8	.999

*NT: Normoxia training group; HT: Hypoxia training group*

*SpO2 (%): Blood oxygen saturation*

*HR: Heart rate*

*RPE: Rate of perceived effort*

*††p < 0.01. Significant difference between groups*

Table 3 shows the results obtained in functional fitness tests, fear of falling and body composition assessment. After the intervention, both in normoxic and hypoxic conditions, a significant increase was observed in the Chair Stand (+ 8.6%∆, ES: 0.46 for NT; and + 19.7%∆, ES: 1.30 for HT), in the Arm Curl (+ 18.4%∆, ES: 1.00 for NT; and + 16.9%∆, ES: 0.94 for HT) and in the 6 min. Walk (+ 5.4%∆, ES: 0.40 for NT; and + 4.7%∆, ES: 0.39 for HT). In CON there was no significant change, with the exception of a decrease in 6 min. Walk (− 5.0%∆, ES: 0.48). HT showed significantly higher levels than CON in Chair Stand (*p* = 0.035) and in 6 min. Walk (*p* = 0.048) after the training program, without observing differences with respect to NT. In connection with values obtained in the FES-I, after the intervention a significant decrease was found in both NT (− 18.6% ∆, ES: 1.17) and HT (− 12.5% ​​∆, ES: 1.11). Additionally, the results observed in NT were significantly lower (*p* = 0.037) than those of the CON, although without differences compared to HT. Finally, there were no significant variations in body composition between pre- and post-values in both training groups (NT and HT). However, CON showed a significant decrease in lean body mass (− 3.1% ∆, ES: 0.13) and a significant increase in fat mass (+ 6% ∆, ES: 0.27), without any between-group differences.

**Table 3:** Variables of functional fitness, fear of falling and body composition before and after the intervention (mean ± SD)

	Control				Normoxia training				Hypoxia training				
	Pre	Post	*95%CI for difference*	*p*	Pre	Post	*95%CI for difference*	*P*	Pre	Post	*95%CI for difference*	*p*	*Interaction effect* *F (p value)*
Chair stand (rep)	12.3 ± 2.2	12.1 ± 1.5	−1.08-0.58	0.547	11.7 ± 2.7	12.8 ± 2.2	0.21–2.01	0.016*	11.2 ± 2.0	14 ± 2.3	1.74–3.79	0.001*†	**10.613 (0.001)**
Arm curl (rep)	15.1 ± 4.2	15.5 ± 5.0	− 1.10-2.01	0.562	14.2 ± 3.1	17.4 ± 3.5	1.55–4.91	0.001*	14.3 ± 3.1	17.2 ± 3.3	0.99–4.84	0.004*	**3.573 (0.036)**
6 min. Walk (m)	554.0 ± 72	525.9 ± 49	−47.47--8.91	0.005*	540.2 ± 66	571.0 ± 90	9.93–51.75	0.005*	562.3 ± 72	590.6 ± 76	4.37–52.19	0.021*†	**10.979 (0.001)**
Sit and reach (cm)	0.1 ± 5.7	1.6 ± 4.6	− 0.08-3.08	0.063	1.1 ± 4.4	2.8 ± 4.3	− 0.015-3.42	0.058	2.0 ± 1.5	2.8 ± 6.7	− 1.19- 2.73	0.436	0.279 (0.758)
Back scratch (cm)	−15.7 ± 7.6	−16.5 ± 11	− 7.06-5.46	0.798	− 6.0 ± 16	− 9.0 ± 9.4	−9.79-3.79	0.379	−17.7 ± 11	− 11.1 ± 13	−1.12-14.42	0.092	1.890 (0.162)
8 ft. Up and go (s)	5.9 ± 0.9	5.7 ± 0.8	− 0.39-0.16	0.404	5.8 ± 0.9	5.6 ± 0.7	− 0.51-0.09	0.174	5.8 ± 0.8	5.6 ± 0.5	− 0.49-0.20	0.405	0.100 (0.905)
SLS (s)	28.0 ± 15.5	23.4 ± 15.4	−10.33-1.14	0.114	21.1 ± 13.3	20.9 ± 12.2	− 6.44-6.00	0.943	34.1 ± 12.1	32.8 ± 12.7	− 8.38- 5.85	0.722	0.592 (0.557)
FES-I	8.3 ± 1.7	8.4 ± 1.5	−0.70-0.80	0.894	9.1 ± 2.0	7.4 ± 0.7	−2.52--0.89	0.001*†	8.5 ± 1.0	7.4 ± 0.8	−2.01--0.14	0.025*	**5.243 (0.009)**
Lean mass (Kg)	43.3 ± 10.0	42.0 ± 10.2	−20.69--5.97	0.001*	46.9 ± 10.6	47.1 ± 11.2	− 6.43-9.53	0.698	47.5 ± 11.7	47.6 ± 12.3	− 8.78-9.46	0.941	**4.646 (0.014)**
Fat mass (kg)	23.8 ± 5.6	25.3 ± 6.0	7.03–23.61	0.001*	25.5 ± 10.1	26.4 ± 10.7	1.28–18.26	0.083	25.4 ± 8.7	25.6 ± 8.7	− 8.76-11.79	0.768	2.218 (0.120)

*SLS: Single leg stance test; FES-I: Short Falls Efficacy Scale-International*

*. Significant differences within-group (Pre-Post)

*†. Significant differences compared to control group*

## Discussion

The aim of the study was to evaluate the effect of 24 weeks of moderate hypoxic strength training on functional fitness, balance and fear of falling in healthy older adults. The hypothesis was fulfilled since the results have shown that the hypoxic training led to an improvement in strength and aerobic endurance, as well as to a lesser concern of suffering a fall, without observing a worsening of the balance. Additionally, in CON group which did not perform any type of strength training, an increase in fat mass and a decrease in lean body mass were observed.

Sarcopenia and loss of muscle strength during aging cause a decrease in physical fitness, functional capability, and balance ability [15]. Strength training has been consistently shown as an effective measure to mitigate muscle weakness, physical frailty, and functional capacity [7, 32]. In the present study, the results obtained confirm these statements since the older adults who performed strength training with elastic bands and kettlebells in normoxia improved the values ​​of the chair stand, arm curl test and 6-min walk tests. Likewise, after the normoxia training program and compared to CON, there was less concern among the older adults to fall. The risk of falling in the older adults is linked to sarcopenia and balance ability [33], so an improvement in muscle strength would be logically associated with a decrease in the risk of falls and the fear of falling [34, 35].

Similar results were observed after the resistance training program in hypoxic conditions, even showing a significant difference compared to CON in the values ​​obtained in the chair stand and in the 6-min walk. These tests were originally designed to assess upper body strength and endurance resistance, being capable of detecting performance differences between those with high and low self-reported physical activity [28]. Previous studies have also showed improvements in physical fitness (chair sit-to-stand) and lung function in older men after a combined training of aerobic exercise and elastic resistance exercise performed for 12 weeks at 3000-m normobaric hypoxia (FiO2 = 14.5%) [16]. A group of people over 65 years improved peak aerobic power and the time to exhaustion after a program that combined aerobic training with passive hypoxic exposures during 90 min three times per week [36]. Similarly, several studies have concluded that an additional intermittent hypoxic training combined with physical exercise augmented the positive effects of exercise on quality of life in older adults [18, 36]. Even a promising treatment for older adults that combines hypoxic periods with moderate hyperoxic periods (30–40% oxygen) has been proposed as a training with more beneficial effects on functional and cognitive capacity than hypoxic conditioning alone, justifying it in a faster recovery of oxygen desaturation and a faster membrane-stabilizing effect in cells of the heart, liver, and brain [37, 38]. In contrast, one study found no significant differences in the maximal isokinetic strength and cardiopulmonary capacity between older people who performed resistance training under normobaric hypoxia compared to those who trained under normoxia. Unlike aforementioned studies, the short duration of this training program (5 weeks) and the low load intensity (25–40% of 1RM) would explain the difference in results between studies. The improvements in the physical performance of the older adults could be explained by specific muscular adaptations to moderate hypoxic conditioning, such as the increase in the recruitment of muscle fibers, the stimulation of muscle protein synthesis, the regulation of the inflammatory response, the efficiency of metabolic oxidation and mitochondrial functioning [15, 39, 40]. Additionally, it should be noted that in our research, the improvement in functional fitness of the older adults who trained in hypoxia did not cause a loss of balance, and it was associated with a decrease in concern about falling. Previous studies have shown that exposure to hypoxia conditions worsened parameters of standing balance [24]. However, in the present study no loss of balance was observed in the SLS test. In this sense, it has been stated that alteration in postural control is larger in hypobaric than in normobaric hypoxia, suggesting that hypobaria instead of hypoxia per is the main factor that causes an altered balance [41]. It has also been stated that the decrease in standing balance is greater the higher the simulated altitude for normobaric hypoxia, with significant differences being observed from approximately 2500 m [23]. Given that our strength training in hypoxia was carried out at a simulated moderate altitude of 2500 m, it seems that arterial oxygenation at that altitude did not influence the processing of sensorimotor signals responsible for balance.

Regarding body composition, there was no significant change in the training groups, although there was a decrease in lean body mass and an increase in fat mass in CON. This maintenance of lean body mass in the hypoxia training group could indicate that the combination of strength exercises with exposure to stressful hypoxic environments could serve to attenuate the loss of muscle mass in the older people [14]. Previous studies have concluded that hypoxic training had an additive effect on weight loss and body composition in older adults [16]. However, these results regarding lean body mass should be interpreted with some caution. On the one hand, there is a consensus opinion that during aging muscle health must be evaluated in terms of muscle mass, strength and functional capacity, rather than just size or lean body mass [29, 42]. On the other hand, there is no unanimity regarding the benefits of hypoxic conditioning on body composition, since factors such as the degree and severity of hypoxia, as well as the duration, intensity, repetitions and recovery of training, play an important role in long-term muscular and metabolic adaptations [43, 44].

The study had some limitations. The experimental design did not have a sham group to analyze the independent effect of only hypoxia exposure. The diet was not monitored during the intervention, as only a record of caloric intake was carried out at the beginning of the programme. Even though participants were instructed not to change their nutritional habits, some dietary habits could have changed during the duration of the intervention. Additionally, the use of other imaging techniques (magnetic resonance and computed tomography) to evaluate the muscle cross-sectional area and regional skeletal muscle, and the isokinetic dynamometer to analyze the rate of force development and the peak torque would have provided more complete information about the functional capacity and muscle quality (strength per unit tissue).

## Conclusions

In conclusion, the findings show that moderate hypoxic conditioning improve the functional fitness and reduce the fear of falling in healthy older adults, without observing a decline in the balance. Likewise, this study provides promising data on the usefulness the hypoxic conditioning in slowing sarcopenia development and loss of strength during aging. Based on the results obtained, moderate physical conditioning in hypoxia is well tolerated by older people, producing a synergistic effect between the benefits of strength training and the benefits of exposure to moderate normobaric hypoxia.

## Data Availability

The datasets used and/or analysed during the current study are available from the corresponding author on reasonable request.

## Abbreviations

ANOVA: Analysis of variance
Asl: Above sea level
BMI: Body mass index
CON: control group
ES: Effect size
FES-I: Short Falls Efficacy Scale-International
FiO2: Fraction of inspired oxygen
HR: Heart rate
HT: Hypoxia training group
NT: Normoxia training group
PAR-Q: Physical activity rating scale
RM: Repetition maximum
RPE: Rate of perceived effort
SFT: Senior Fitness Test
SLS: Single Leg Stance test
SpO2 (%): Blood oxygen saturation
